# Letter to the Editor for the article “Laparoendoscopic single site myomectomy versus conventional laparoscopic myomectomy for uterine myomas: a systematic review and meta-analysis”

**DOI:** 10.1097/JS9.0000000000003253

**Published:** 2025-08-22

**Authors:** Deyu Zhang, Yingfang Zhou, Chao Peng

**Affiliations:** Department of Obstetrics and Gynecology, Peking University First Hospital, Beijing, China

*Dear Editor*,

We were interested in reading the impressive study by Xie W *et al*^[1]^ in the *International Journal of Surgery* and would like to congratulate the authors for their interesting findings. While the study provides valuable insights into the perioperative outcomes and cosmetic advantages of laparoendoscopic single-site myomectomy (LESS-M), we believe two critical issues warrant further discussion: hidden blood loss (HBL) and the impact on fertility. These aspects are crucial for clinical decision-making but were not sufficiently addressed in the original analysis.

## The first issue pertains to hidden blood loss (HBL): an overlooked but clinically significant factor

While HBL is well-established in orthopedic surgery, its significance remains underappreciated in gynecological procedures^[2]^. However, emerging evidence suggests that HBL may be significantly higher in LESS-M compared to conventional laparoscopic myomectomy (CLM). For instance, Zhang *et al*’s retrospective cohort study demonstrated that patients undergoing LESS-M had a visible blood loss (VBL) of 115.4 ± 180.6 mL and an HBL of 364.3 ± 252.6 mL, accounting for 74.4% ± 22.4% of the actual total blood loss (TBL). In contrast, CLM patients had a VBL of 187.9 ± 198.5 mL and an HBL of 306.8 ± 304.7 mL, constituting 58.9% ± 30.2% of the actual TBL^[3]^. Based on these findings, we recommend that future meta-analyses incorporate standardized HBL assessment (e.g., using the Gross formula) to provide a more comprehensive evaluation of surgical blood loss.

## The second crucial point concerns the impact on fertility

Given that a significant proportion of patients undergoing myomectomy are of reproductive age, the omission of discussing the fertility impact associated with the two techniques is a notable gap. Available data indicate a pregnancy rate of 66.7% after LESS-M compared to 50.0% after CLM. Notably, LESS-M was also associated with a higher term delivery rate (66.7% vs. 58.3%) and a shorter time to conception (7.6 months vs. 10.1 months)^[4]^. These findings suggest differing impacts on fertility outcomes between the surgical approaches. Consequently, we propose that future systematic reviews incorporate fertility-related outcome measures (such as pregnancy rate, time to conception, and obstetric complications) to better inform patient counseling.

We commend the authors for their rigorous analysis and hope these suggestions will be considered in future updates to the analysis or related research endeavors.

In compliance with the TITAN Guidelines 2025 on the declaration and use of artificial intelligence (AI), we would like to highlight that our discussion in this letter was informed by available data and evidence, and no AI tools were used in the drafting of this manuscript^[5]^.

## Data Availability

This manuscript is a comment. All the data from the current study are publicly available.

## Research registration unique identifying number (UIN)

Because this is a commentary of a research paper, research registration is not required.
